# The manipulation of odor availability of training aids used in detection canine training

**DOI:** 10.3389/falgy.2024.1445570

**Published:** 2025-01-07

**Authors:** Katylynn B. Sloan, Michele N. Maughan, Caitlin E. Sharpes, Robin R. Greubel, Shawna F. Gallegos, Aleksandr E. Miklos, Lindsay D. Waldrop

**Affiliations:** ^1^ Advanced Research and Capabilities Division, United States Secret Service, Washington, DC, United States; ^2^ Precise Systems, Inc., Lexington Park, MD, United States; ^3^ K9Sensus Foundation, Lucas, IA, United States; ^4^ Oak Ridge Institute for Science Education (ORISE), Oak Ridge, TN, United States; ^5^ Applied Synthetic Biology and Olfaction Branch, U.S. Army DEVCOM Chemical Biological Center, Aberdeen Proving Ground, MD, United States; ^6^ Schmid College of Science and Technology, Chapman University, Orange, CA, United States

**Keywords:** canine, odor movement, scent detection, training aids, dispersion, dog training, olfactory science

## Abstract

Detection canines can identify numerous substances for which they have been trained. Historically, and a point of ongoing contention, detection canine threshold (i.e., sensitivity or limit of detection) training has primarily focused on changing the weight of the training aid substance used. There has been minimal focus on other principles, such as surface area, confinement, and temperature, which can be manipulated to affect odor availability. That said, trainers have been manipulating odor availability for years without necessarily understanding the governing scientific principles. The aim of this review is to highlight the principles that control odor availability of a substance and how an end user can apply these principles for operational detection canine training needs.

## 1 Introduction

While detection canines have been successfully deployed for generations, the related scientific backing for how canines are able to accomplish their intended task is relatively new and, in some cases, still unknown (1). The Organization of Scientific Area Committees (OSAC) in conjunction with the American Academy of Forensic Science (AAFS) Academy Standards Board (ASB) are working to develop national consensus standards for the forensics sciences which includes detection canines baseline protocols and guiding principles for a detection canine program. The published ANSI/ASB STD 092: Standard for Training and Certification of Canine Detection of Explosives requires that the canines shall be exposed to varying concentrations/amounts of available odor, yet repeatedly mentions minimum weights of material to be used within training and certification scenarios (2). While material weight is only one factor contributing to odor available for canine detection, it is one of the only factors that can be directly quantified and controlled in the field or operational use, making it an ideal candidate to reference in a standard.

While technically possible, it is unrealistic and impractical for a canine trainer to know the exact concentration of available odor for canine detection (3, 4). It is critically important for trainers to train with different concentrations of available odor to ensure that their canines are proficient in detecting a wide range of target odors likely to be encountered operationally, yet it's difficult for them to know when they are accomplishing this during training. This does not mean the relative odor concentration cannot be modulated to improve the range of odor concentrations that canines detect. Canine trainers and handlers have routinely manipulated odor concentrations through practices developed via trial and error over decades, often without realizing the practice works because they are capitalizing on features of the Ideal Gas Law, Clausius-Clapeyron equation, and the laws of diffusion, etc. which scientists have been studying for centuries (5, 6).

**Figure 1:**
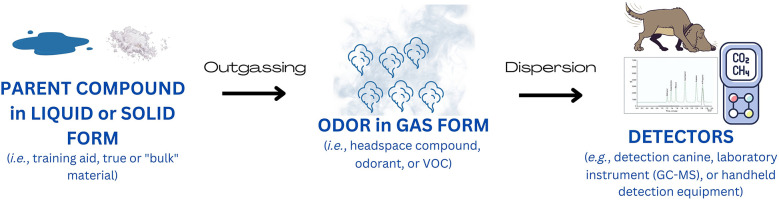
The journey of an odorant from source to sensor. In this example, the source material (also known as the training aid, true material, or “bulk” material) is emitting its odor, or odorants, via outgassing. Once the odorant is in its gaseous form, the molecules then move from the source to the sensor by dispersion (i.e.*,* spread). Dispersion allows the odor to travel from the source to the sensor (i.e., detector) to be made available for analysis. Typical sensors are detection canines, laboratory instruments (gas chromatography-mass spectrometry), and handheld detectors.

Here we review the critical physical principles in alignment with the canine detection community's terms and definitions; these principles must be understood and employed for a rigorous detection canine training program to succeed. Within this review, we aim to unite the anecdotal practices developed by canine trainers and handlers with scientific principles guiding odor concentration modulation practices. Several commonly encountered questions from the detection canine training community to the scientific community have been conglomerated and answered resulting in general guidance for increasing or decreasing odor availability. The overall journey of an odorant from a source to a sensor is depicted in Figure 1, with greater detail provided throughout this review to describe the mathematical, chemical, and physical laws governing odor availability. A few points should be noted pertaining to the scope of this review.

• We use the terms “odor” and “scent” somewhat interchangeably, however, the ANSI/ASB standard defines odor as the volatile chemicals emitted from a *substance* and scent as the volatile chemicals emitted from a *live human* (7).
• We use the term “training aid” as defined by the OSAC standard as “target odor/scent sources used for training”. Training aids can encompass true material and alternative training aids (such as sorption, mimic, dilution, and vigilance aids) (8, 9) for any canine detection discipline.
• We focus on explosive training aids in our examples due to the abundance of available research whereas peer reviewed information for other detection disciplines is significantly limited. The vapor pressures and headspace composition of many explosives have been studied and published; thus, they make the best examples when discussing odor availability.
• The principles of odor availability apply regardless of the type of training aid. While some of the values (e.g.*,* vapor pressure) and parameters (e.g*.,* temperature) may differ between training aids, the laws of physics and math determine how gases behave.

## 2 Foundational physical principles governing odor availability

Once the training aid is set out, odorant must be liberated from a solid or liquid state into a gas or vapor, by the complex process of outgassing. The odor must then make it out into environmental air by permeating any container surrounding the training aid. Odor is then transported in air away from the source, where it can be sampled by the canine (Figure 1). At each of these steps, the amount of odor available to a detection canine can be manipulated during training.

However, prior to discussing the manipulation of training aid odor availability, the foundational scientific principles that govern odor availability of a substance must be discussed. Scientists use many words that have common definitions more formally, using strict definitions with very specific meanings. This disconnect in language creates confusion and often represents a significant barrier to communication between the scientific and practitioner communities. The aim of this section is to provide an overview of the principles and codify the terminology (Table 1) used by the canine training and scientific communities to describe the same phenomena. Each subsection describes the physical processes involved in each step of the process described above.

**Table 1:** Convergence of scientific terms within chemistry and physics with the terms and definitions of the canine detection community (10).

Physics & chemistry terms	Definition	Canine detection community terms
Adsorption	Dissolved/gaseous substance that binds to the surfaces of other materials in the surrounding area	Residual odor, contamination, “stickiness” of an odor
Advection	Transport of dissolved/gaseous substance by bulk fluid movement	Odor movement over a distance, odor plume, scent cone, vapor wake
Concealment	Emplacement of a training aid in a training scenario that prevents the canine team from visually locating the training aid	Hide
Concentration gradient	The gradual change in concentration of a substance between regions of high and low concentrations	Scent cone, odor plume
Condensation/deposition	State transition of mass/matter from gas to liquid/solid	Sweating (e.g., the training aid is forming water around it when moved from cold to hot environments or vice versa), recrystallization
Contamination	When an odor/scent is inadvertently introduced. Contamination can include the following: contamination of a search area with a target odor/scent or contamination of a target aid with competing odor/scent	“Dirty”, contaminated, cross-contaminated
Diffusion	Spreading of a dissolved/gaseous substance via random, molecular movements	Odor movement (directly off the target substance)
Dispersion	The complete process that spreads or transports mass away from the source material into the environment (combination of advection and diffusion)	Odor movement (directly off the target substance), odor plume, scent cone, vapor wake
Flux	The rate of flow of a fluid (gas or liquid) or particles across a given area	Flow, movement
Gas/vapor	A state of a substance that has no defined shape or volume, but expands to fill its container/a substance that exists as a mix of states (gas, liquid) at room temperature	Odor, odor profile (collection of odorants), odorant (molecule), scent, smell
Headspace	The air directly above a material	Atmosphere, area around the training aid, volume above a solid or liquid within a closed container
Mass	Amount of matter	Amount of true or bulk material. Often used to describe the weight of a training aid
Mass flux	The rate at which a substance sublimes/evaporates as mass per unit of area per unit time	Permeation, permeation rate
Odor	The cognitive process by which a person defines or names an odor profile	Odor, odor profile (collection of odorants), odor signature, odorant (molecule), scent, smell
Odorant/headspace component or compound/Volatile organic compound (VOC)	Individual chemical vapor in the headspace of a substance	Odor, odor profile (collection of odorants), odorant (molecule), scent, smell, volatile organic compound (VOC), odorant
Outgassing	The release of gas (odor) from a material via mechanisms such as: • sublimation* • evaporation* • bulk diffusion • permeation • vaporization • desorption—of previously adsorbed molecules • seepage from cracks and seams • slow chemical reactions that form gaseous products	Off-gassing
Permeation	The rate at which a substance passes through a barrier due to diffusion as mass per unit area per unit time	Permeation, permeation rate
Scent	Volatile chemicals emitted from a live human that are perceived by the canine through olfaction. “Scent” has traditionally referred to canine detection of humans. “Odor” has traditionally referred to canine detection of a substance	Odor, smell, bouquet
Sublimation/Evaporation	State transition of mass/matter from solid/liquid to gas	Off-gassing, out-gassing
Turbulent mixing	Process by which unsteady flow equalizes concentrations of mass in time and/or space	Odor movement (away from the target substance)
Vapor pressure	The pressure exerted by a vapor on its surroundings. It is a measure of the tendency of a material to change from a solid/liquid into a gas	Off-gassing, out-gassing, volatility
Wake	Region of slow flow and lower relative pressure on the trailing edge of flow behind an object	Odor pooling area

*Predominant mechanisms by which true material canine training aids outgass.

For the purposes of this review, containment describes the packaging of the training aid for usage, storage, and transport. Primary containment is the immediate packaging that surrounds the training aid. Secondary containment is used to store the training aid within primary containment when not in use. The goal of secondary containment is to minimize contamination via an impermeable container. Some organizations utilize multiple layers of containment to further reduce the likelihood of contamination. Common secondary containments include glass jars with sealing metal lids, metallic lined bags, plastic storage containers, etc. Tertiary containment describes the large transport case containing all the training aids in secondary containment. The goal of tertiary containment is for transport or long-term storage and typically consists of a hard case to minimize damage of the training aids.

Since most training aid odors have a chemical profile consisting of multiple chemical compounds, it is worth considering how dogs interpret information in the headspace of an aid, or its **odor signature** (also commonly referred to as the **odor profile**). It is commonly said that a detection canine is trained to detect the **odor** of nearly anything, and they are often described as a “black box technology” since the response induced by the target odor, and its individual odorants, varies between canines (9, 11). **Odorants** are molecules that are properties of the external world objectively defined in terms of their physical and chemical characteristics and capable of being transposed by particular nervous systems into odors (10).

Compounds within the odor profile of a target material continue to be studied for the development of training aids and attempts at better describing canine olfaction. Previous studies, all be it with significant knowledge gaps of certain training aids, concluded that there are numerous odorants associated with a single training aid due to the manufacturing process (solvents, taggants, stabilizers, degradation products, adulterants, cutting agents, etc.), and decomposition of the training aid. For these reasons, training aids are more often than not a mixture (9, 12–16). Additionally, contaminants resulting from manufacture, storage, and operational use may also be found within the headspace adding to the odor signature used by canines; however, these two topics are outside the scope of this review and have been presented elsewhere (12, 17–25).

### 2.1 Odor generation

Canine training aids are typically solid or liquid substances contained within a permeable barrier/membrane material (primary containment). It is essential to remember that detection canines are not necessarily detecting the physical substance (i.e., the solid or liquid substance), they are detecting the gas phase (vapor) of the target material which may include the target material and/or other odorants resulting from synthesis and degradation processes (9). The vapor may consist of solely gaseous particles, or a mixture of gaseous particles and aerosolized solid or liquid particles. For the purposes of this review, the terms vapor and gas are used interchangeably, and we do not differentiate between aerosolized particles and gaseous molecules as both are detectable by canines (26).

Odor is emitted from training aids through a multi-factorial phenomenon called **outgassing**. Outgassing is, at its most simplistic, the release of gas molecules, however, it is a complex process that includes desorption, diffusion, permeation, sublimation, evaporation, and vaporization (27, 28). During the creation or manufacture of a training aid, gas molecules were dissolved, trapped, frozen, or adsorbed inside the training aid. Since canine training aids can consist of true material, an alternative training aid, a liquid or a solid, there are a wide variety of substrates from which training aid odor outgasses. There are phase transitions, such as **evaporation** and **sublimation**. The former occurs when a liquid transitions into a gas, whereas the latter occurs when a solid directly transitions from a solid to a gas. The opposite of evaporation is **condensation**, where a gas transitions into a liquid, and the opposite of sublimation is **deposition**, where a gas transitions into a solid. **Permeation** occurs when training aid odor travels through a substance before being released into the environment (e.g., a training aid contained in a permeation bag). **Desorption** is the opposite of adsorption and occurs at the surfaces of materials whereby previously adsorbed gas molecules are released from the training aid back into the surrounding environment (e.g., an odor “soak” or Getxent™ tube). Diffusion, discussed in greater depth later, is the movement of molecules from areas of high concentration to low concentration.

It should be noted that during this process, condensation and deposition are occurring simultaneously as are their evaporation/sublimation counterparts, though at a lesser rate. The rate at which the training aid sublimes/evaporates minus the condensation/deposition per unit of area is **mass flux**. Mass flux will continue to occur until the surrounding environment is saturated with gas molecules. At this point, the material is said to be at **equilibrium** because the rate of sublimation/evaporation equals the rate of deposition/condensation. If the environment cannot be saturated (as in the case of an open/outside area), mass flux will continue to occur until all the source material has been depleted. Equilibrium, for all practical purposes, can only be achieved in a closed system/container (Figure**)**.

**Figure 2:**
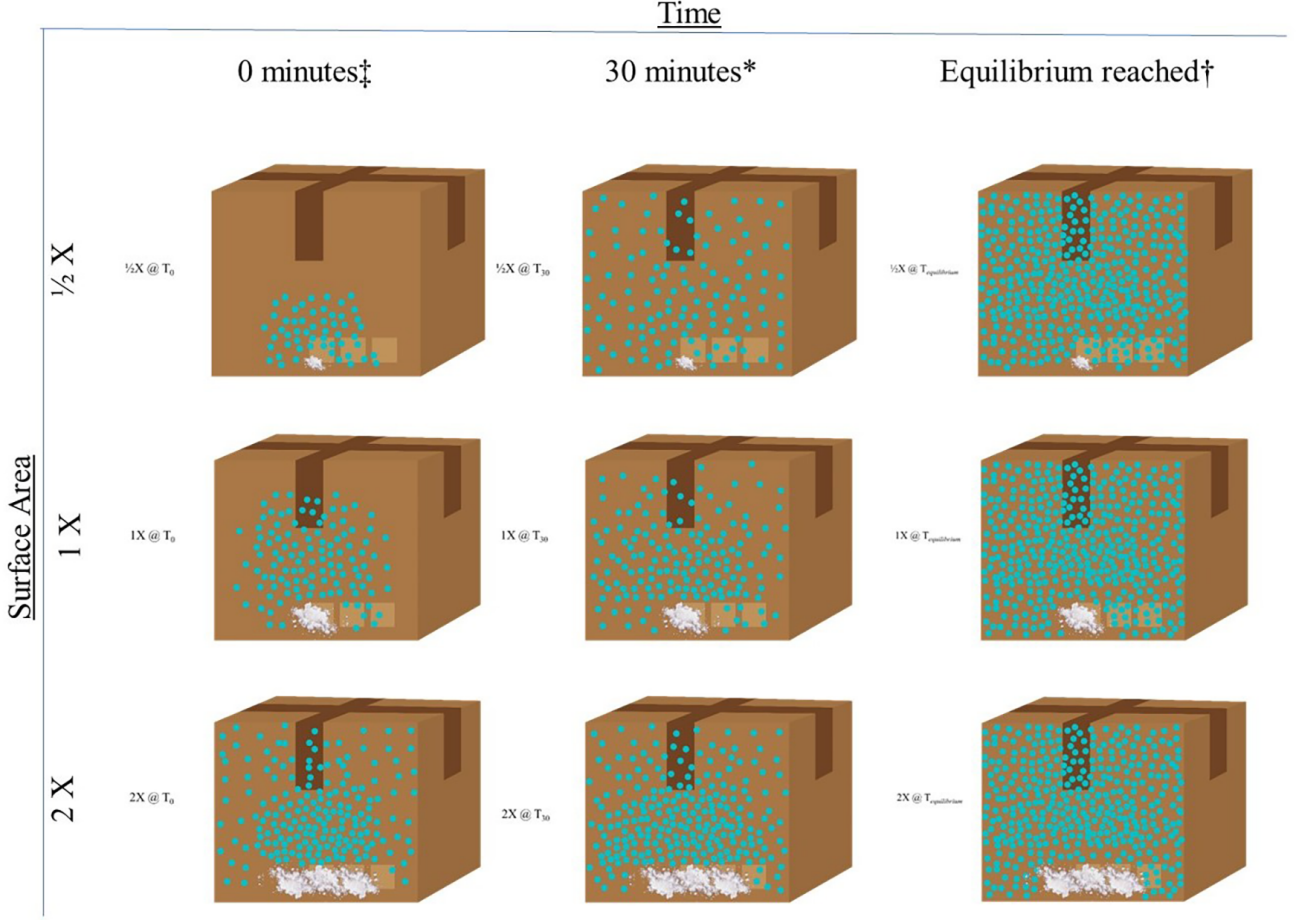
Relationship between surface area and time on the diffusion of training aid odor in a closed system. Whereby X is the surface area (SA) of the training aid (TA), the number that proceeds the X is the multiplier that either increases or decreases the SA. TA. ^‡^Time = 0 (T_0_) represents when the TA is first placed in the hide location. *Time = 30 min (T_30_) represents the typical “set time” for the training aid employed by the canine detection community for certifications. ^†^Time = equilibrium reached (T_equilibrium_) represents the amount of time **(T)** it takes for the headspace around the TA (e.g., the box in this example) to become saturated. In this example, this amount of time will be shortest for the 2× surface area TA and longest for the ½× surface area.

Figure 2 illustrates the scenario in which a training aid is placed inside a closed system (e.g., a cardboard box as a hide location for a canine search) and the relationship between the surface area of the training aid [e.g., spread out amounts of 50 g (1/2×), 100 g (1×), and 200 g (2×)], and time (e.g., 0 min as soon as the training aid is placed in the box, 30 min after a “standard” set time, and the time at which equilibrium is reached which will be different for each training aid based on the surface area of the training aid when all other conditions are the same (pressure, volume, and temperature). Once equilibrium is reached, the ½×, 1×, and 2× training aids all have the same amount of odor in the box.

As discussed by Giordano et al.*,* on the low end of sample weight (tens of milligrams), the concentration of available odor is reduced from that of expected equilibrium concentrations (29). While previously stated that the amount/weight of the material does not impact the concentration of available odor at equilibrium, this is caveated that there is sufficient material that equilibrium can be achieved. While definitive minimum weights of material are a current knowledge gap, the ability for a training aid to reach equilibrium is a function of the surface area of the material in relation to the volume of the closed container it is contained within. As a general rule, if there is visible [gram(s)] amounts and/or the bottom of the primary concealment is 100% covered by material, obtaining equilibrium is highly likely (29).

The concentration of odor at equilibrium is directly related to a chemical's **vapor pressure**, referring to the fraction of total pressure that is due to the chemical's vapor. Most simplistically stated, vapor pressure describes the willingness of a substance to release its odor to the atmosphere. Chemicals with a higher vapor pressure will release more mass into the air at equilibrium compared to those with lower vapor pressures in similar situations. This relationship can be explained by the ideal gas law. The **ideal gas law** describes the relationship between the pressure, volume, and temperature of a gas to approximate its behavior. Due to this relationship, the vapor pressure of a training aid is temperature dependent (Table 2), and unfortunately, the dependence is not linear as the vapor pressure grows roughly exponentially as temperature increases.

**Table 2:** Select vapor pressures and saturated headspace concentrations (6, 30–32).

Compound		Molecular weight (g/mol)	Vapor pressure (atm @ 25°C)	Saturated headspace concentration at equilibrium (ppm)	Saturated headspace concentration at equilibrium (g/L)
NG	Nitroglycerin	227	6.45 × 10^−7^	0.645	5.98 × 10^−6^
TNT	2,4,6-Trinitrotoluene	227	9.15 × 10^−9^	9.15 × 10^−3^	8.49 × 10^−8^
AN	Ammonium nitrate	80	1.47 × 10^−8^	1.47 × 10^−2^	4.80 × 10^−8^
PETN	Pentaerythritol tetranitrate	316	1.07 × 10^−11^	1.07 × 10^−5^	1.38 × 10^−10^
RDX	Cyclotrimethylene trinitramine	222	4.85 × 10^−12^	4.85 × 10^−6^	4.40 × 10^−11^
AP	Ammonium perchlorate	117.5	4.01 × 10^−14^	4.01 × 10^−8^	1.92 × 10^−13^
Cocaine		303	2.51 × 10^−10^	2.51 × 10^−4^	1.11 × 10^−9^
Heroin	Diacetylmorphine	369	9.99 × 10^−13^	9.99 × 10^−7^	1.51 × 10^−11^
MDMA	N-Methyl-3,4-methylenedioxyamphetamine	193	3.64 × 10^−6^	3.64	2.88 × 10^−5^

The saturated headspace concentration at equilibrium data helps demonstrate the differences amongst the selected compounds, for example, the large amount (ppm) of nitroglycerin compared to the small amount (ppm) of ammonium perchlorate. The direct relationship between vapor pressure and odor concentration is also demonstrated with the higher the vapor pressure, the higher the saturated headspace concentration. For example, MDMA's vapor pressure is comparatively high (3.64 × 10^−6^) and therefore corresponds to the relatively large amount (ppm) of MDMA odor in the headspace (3.64).

Many materials that canines are trained to detect are composed of more than one chemical compound, potentially a complex mixture, each with their own physical properties such as vapor pressures and evaporation or sublimation points. When the training aid is exposed to a volume of air (like that of a primary container), all components of the material will begin to fill the air according to these physical properties. The air inside this container is referred to as the **headspace**, and after equilibrium, this headspace will contain a characteristic combination of vapors. Each component in the headspace is referred to as an **odorant**. It should be noted that the headspace can contain highly volatile and semi- or low-volatility compounds, all of which may or may not be perceived by the canine and considered part of the odor signature for canine olfaction.

Even though all components of the training aid will reach equilibrium between matter being released into and returning from the air within the primary container, the amount or mass of material that is ultimately liberated into the air can differ between chemicals. This relationship is described by Raoult's Law, in which the overall pressure is the sum of the partial pressures from each individual compound. The vapor pressure of individual compounds will help determine the fraction of the total headspace of a specific odorant (33).

Often detection canines encounter a wide variety of training aids targeting a single source or “-based” material. For example, RDX-based explosives are a mandatory odor for detection for an explosives detection canine (2). RDX can be found in multiple forms including pure, or as the main energetic in plastic explosives (e.g., C-4), detonating cord, and other explosive formulations. Case in point, a pure material like cocaine will have the decomposition (i.e., breakdown) product of methyl benzoate odorant in the odor plume and triacetone triperoxide's (TATP) odor profile consists of the single odorant TATP (12, 34). Target materials composed of mixtures, like the explosive Composition 4 (C-4), trinitrotoluene (TNT), and smokeless powders, have significantly more complex headspace compositions. C-4′s odor profile which is a complex mixture of the odorants consisting of the energetic material (RDX), plasticizers (2-ethyl-1-hexanol, cyclohexanone, etc.) and taggant [2,3-dimethyl-2,3-dinitrobutane (DMNB)], and industrial solvents or degradants from the formulation (12).

**Figure 3:**
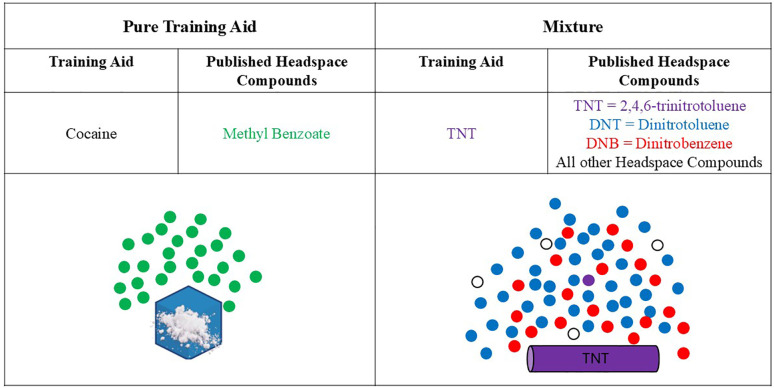
Notional headspace composition images of a pure training aid and a mixture. Note, there is significantly less TNT found in the headspace of the TNT training aid due to competition; DNTs and DNBs are more prevalent. Published headspace compounds for cocaine (34) and TNT (11, 35).

While the exact composition of the “-based” material may be unknown, the proportion of a single odorant is only one of several odorants making up the overall composition. Therefore, the vapor pressure of a pure training aid will always be higher than the vapor pressure of a “-based” mixture training aid (as described by Raoult's Law). Inherently, this is logical because there will be “competition” for each compound to go into the headspace. The concentration of odorants in the odor plume of a mixture training aid will be proportional to the vapor pressure of the odorant, where odorants with higher vapor pressures will have higher concentrations than lower vapor pressure odorants. Figure 3 illustrates this concept for both a pure (e.g.*,* cocaine) and a mixture training aid (e.g.*,* TNT), whereby the simplicity of the pure training aid is clear by the single headspace compound while the mixture training aid odor is comprised of numerous headspace compounds of differing concentrations.

Note, all of the physical properties discussed thus far have not addressed the amount (weight) of the training aid (solid or liquid) impacting the vapor pressure or concentration of odorants in the headspace. That is because the concentration of the training aid odor is constant and at equilibrium, the concentration of the training aid material does not impact the concentration of available odor in the headspace. Since concentration in a solid is calculated as the mass percent, increasing the weight of the training aid will not change the headspace concentration. At equilibrium, the concentration of the training aid material does not impact the concentration of available odor in the headspace because the maximum amount of odor has entered the headspace. More simply stated, weight of the training aid is irrelevant when the training aid is at equilibrium with the environment (e.g.*,* 99% pure cocaine training aid will always have a concentration of 9.9 × 105 ppm at equilibrium regardless of the weight). However, detection scenarios utilizing canines are almost never conducted when equilibrium conditions have been achieved (because equilibrium conditions require a closed system that would, by definition, prohibit odor availability). As such, one can manipulate the available odor in nonequilibrium conditions by changing certain variables (Section 3).

### 2.2 Permeation of odor through primary containment

After entering a gaseous state, odor must leave primary containment into environmental air. Primary containment typically consists of a permeable material that allows for the escape of odor while minimizing particulate loss or leakage. Thus, the goals of effective primary confinement are threefold: first, to prevent physical loss of the material; second, to minimize contamination of the material; and third, to ideally provide a constant and stable source of odor. Common primary containment used in canine training includes bags (e.g., plastic, nylon, duck cloth fabric, or canvas bags), jars, and purpose-built permeable devices such as Controlled Odor Mimic Permeation Systems (COMPS) (36, 37) and the Training Aid Delivery Device (TADD®) (17, 38, 39).

The dominant physical process during this step is diffusion of odor through the barrier provided by the material of primary containment. **Diffusion** is the process by which mass is transported by molecular movements, the random, unguided spreading of mass from areas of high concentration to those of low concentration (40). The diffusivity of a chemical is a property that changes with the state of the chemical, what it is dissolved in, and its size. Diffusivity of a chemical in a solution is typically quantified by the diffusion coefficient (*D*), which is a standardized measurement of area per unit time. If a chemical is diffusing across a solid barrier material, it is typically described as **permeation** (*J*) and the rate at which mass moves from one side to the other of the barrier is a **permeation rate**.

**Figure 4:**
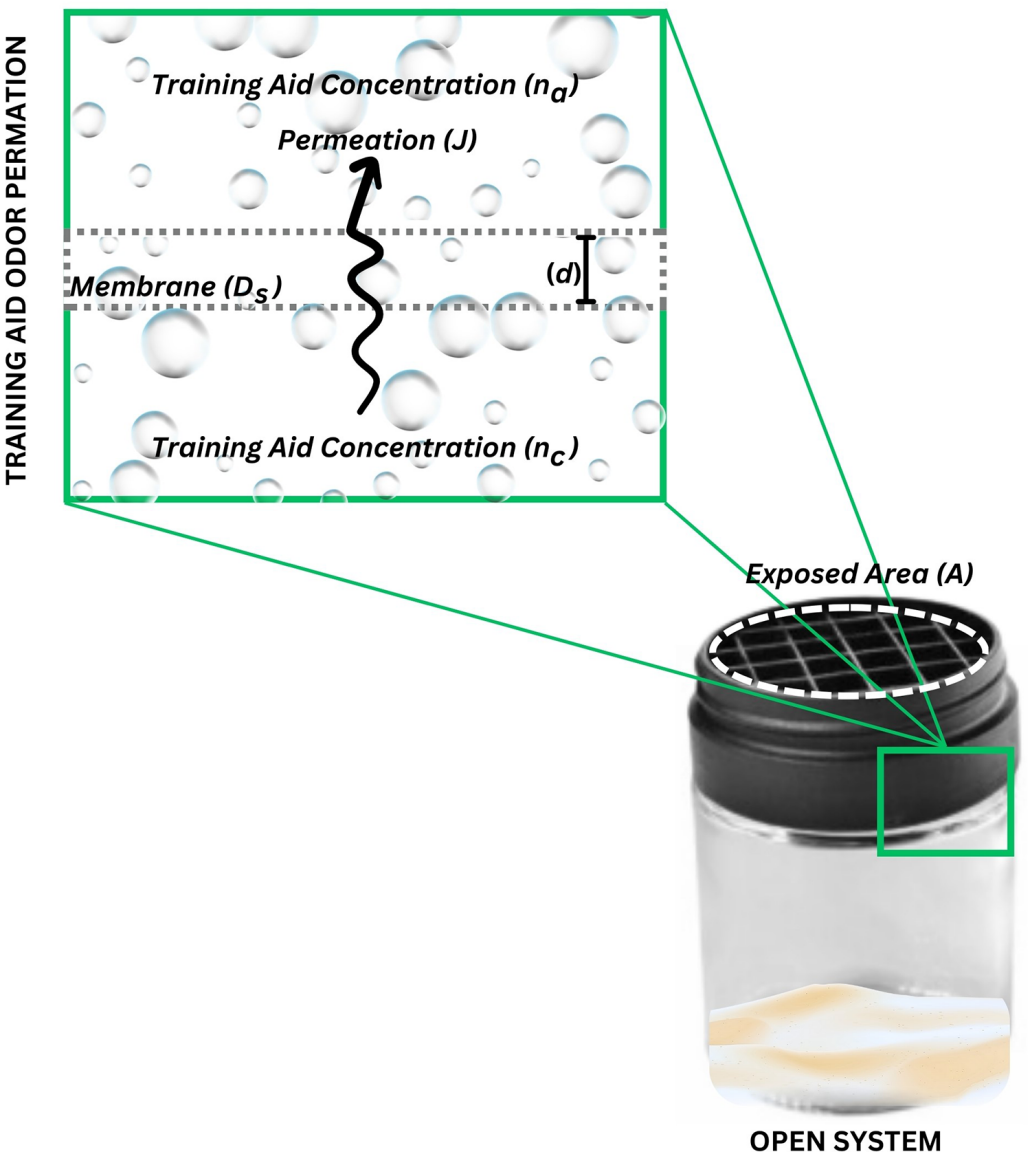
Permeation of odor as described by Dravniks’ equation. This figure illustrates the elements in Dravniks’ equation that govern the principles of odor permeation.

Odorant molecules in the headspace of the primary container will diffuse across the primary containment material, making this odor available to the air outside of primary containment. Once equilibrium or near equilibrium conditions are achieved within the primary containment, the permeation rate stabilizes and can be described by Fick's law, as derived by Dravnicks et al. (41) (Figure 4):

$$J=AD_{s}\frac{n_{c}-n_{a}}{d},$$

where the permeation (*J*) is equal to the multiplication of the exposed area (*A*), by the diffusion coefficient of the odorant with respect to the containment material (*D_s_*), by the concentration of the vapor in equilibrium in the container (*n_c_*) calculated from the vapor pressure minus the actual concentration of the vapor in external air (*n_a_*), divided by the thickness of the primary containment material (*d*). *D_s_* is specific to both barrier material and odor since some materials are notably more permeable than others. While not a perfect estimation of the true permeation due to the assumptions and limitations described by others (28, 42–44), it does provide a simplistic equation in which the manipulation of training aid configuration can be observed.

While outside of the scope of this review, the authors have noted a significant knowledge gap in published values of *D_s_* values associated with detection canine training aids. Historically, in-depth mathematical evaluations of the diffusion, advection, and odor availability of detection canine training aids has not been an area of substantial research and those working in the field default to the estimations used by Dravnicks et al. (41).

Since it takes time for equilibrium to be reached inside the primary container and for odor to permeate the material of the primary container, it is not ideal to use a training aid immediately after placement into primary containment. Odor availability can be low or inconsistent after a short time. This observation has been noted as after the training aid is initially packaged, there will be a period of unstable and changing permeation rate as the training aid material attempts to achieve equilibrium within the primary containment (16, 17, 45–47).

### 2.3 Transport of odor by environmental air flows

**Figure 5:**
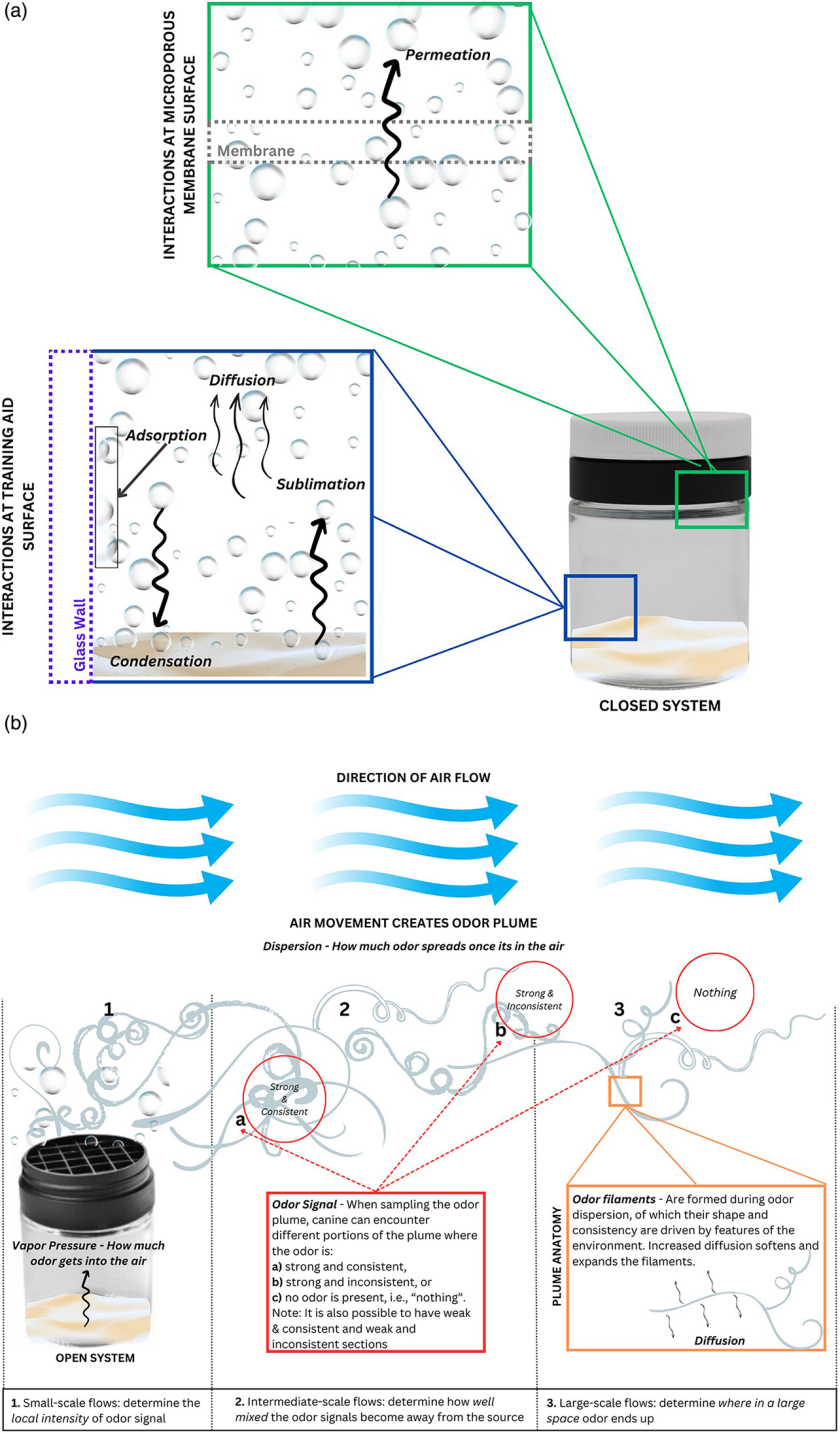
Odor movement within a closed system and throughout an open system. **(a)** In this closed system, illustrated by a TADD®, permeation (green box) is the mechanism by which training aid gases are transported across the microporous membrane. Within the TADD® (blue box), condensation and sublimation occur continuously as the training aid aims to reach equilibrium. There are also surface interactions (adsorption) between the gas and glass jar. Diffusion is the main mechanism by which gas moves within this closed system. **(b)** Once the system is opened to the environment, several processes occur. (1) Permeation determines how much odor gets into the air and enter small-scale flows which determine the local intensity of the odor signal. (2) Intermediate-scale flows then determine how well-mixed the odor signals become as they are transported away from source. (3) Finally, large-scale flows determine where in a large space odor ends up. The odor signal that is available for canine detection (red box) are described by the strength and consistency of the localized odor plume. The plume anatomy (orange box) is composed of odor filaments that can reach far beyond the source material due to air flow. There are two physical processes in plume dispersion: advection (bulk movement) and diffusion. They are perpetual processes and can be described as a ratio of which one is larger or more dominant (the ratio is the Péclet number). Diffusion is dominant in the closed system TADD® because it outweighs whatever miniscule amount of airflow is occurring. Once the TADD® is opened and odor permeates out of the membrane, air flow is significantly larger than the scale of diffusion that advection dominates.

After leaving primary containment and entering the external air, odor is transported by environmental air flows away from the hide in a process called dispersion. **Dispersion** is a combination of diffusion of the odor in air and advection (i.e., transport) of odor due to bulk air flow Figure 5a illustrates many of the simultaneous processes that are occurring in a closed system (e.g., closed TADD® containment) and the processes that dominate odor transport once the odor has permeated through the membrane and entered an airstream of environmental air flows, Figure 5b.

In the closed system, the training aid substance is off-gassing headspace compounds into the surrounding containment. These odorants are simultaneously, in the case of a solid training aid, subliming from the training and condensing back onto/into the training aid. Diffusion allows the odorants to move about the interior of the containment. Meanwhile, some of the odorants are “sticking” or adsorbing to the interior surface of the glass jar and some are permeating through the membrane and into the space between the membrane and the closed cap. In this scenario, the training aid will be able to reach equilibrium within this closed system.

Once the system is opened, i.e., the cap is removed, the odorants are liberated from the TADD® and can now enter the surrounding air. Here, especially, the vapor pressure of the training aid should be considered as this will determine how much odor will be released from the training aid. The higher the vapor pressure, the more odor is released from the training aid. The environmental air flows are discussed in terms of scale with small-scale flows occurring near the training aid and determining the local intensity of the odor signal, intermediate scale flows occurring further away and determining how well-mixed the odor signals are in air flow, and large-scale flows determining where these odor signals end up in the environment. Odor signals are portions of the odor plume where odor can be sampled.

These flows are important to keep in mind when thinking about detection training scenarios and hide locations. It is possible that odor signals are not present where the dog is sniffing despite being close to the training aid. It is also possible that odor signals are present where the dog is sniffing despite being far away from the training aid. We describe these odor signals by their intensity and consistency to better understand the sniffing or sampling area within the odor plume's anatomy. Because odors and dogs are not static in the environment and they are constantly moving within air flows, dogs are given many opportunities to encounter an odor signal. Furthermore, because of the intricate and sophisticated mechanisms by which dogs conduct their olfactory tasks, and their anatomy, physiology, and genetics, they are even better poised to cross paths with odor signals (48–60).

As air flows around the primary container, it will draw out some odor-laden air very near the surface of the container. This action creates high-concentration filaments of odor which are then moved away with air currents far from the position of the primary container **(5B**) (61, 62). Diffusion will act to soften the edges of these odor filaments, transporting odor across small-scale layers of air that otherwise would not mix. These odor filaments vary based on how the air moves and create localized high and low concentrations of odor within the plume that vary in space and time to create the irregular plume structure colloquially known as a “scent cone”. This phenomenon is a key principle that is extensively exploited when “scenting to source” in which a detection canine enters a scent cone of known odor and then follows the plume until the source is found (55). The signals provided by the odor plume vary in strength, time, and space, and how canines use these cues to locate the source is still under active study.

Once odor leaves the source, the concentration of odor experienced by the dog during a search via sniffing is influenced by the movement of air via **turbulent mixing**. Much like stirring creamer into coffee, initial concentrations liberated from the primary container can be mixed with clean air to reduce the effective signal of that odor ultimately experienced by the dog. Several factors can increase mixing: the speed and direction of environmental air flows (e.g., HVAC systems, wind, convective flows), large objects in the environment that disrupt flow, and surface roughness (e.g., smooth glass vs. cobblestone). High air flows and rough surfaces can increase mixing so much that detection canines can struggle to scent to source (63–65).

Large objects and rough surfaces in the environment can also serve to locally concentrate odor signal by preventing turbulent mixing. Objects in flow generate a **wake** at the side trailing oncoming flow, or a region of low relative pressure and flow. Odor filaments can collect here, leading to a relatively higher concentration signal than elsewhere in the immediate environment because the slow flows limit turnover and mixing of the wake. Other types of dead- or low-flow regions may have a similar effect. Furthermore, odor sources close to the substratum (i.e.*,* the boundary layer closest to the solid surface) will tend to concentrate and keep odor signal close to the ground due to the lower flow of air close to solid surfaces. Depending on the placement of the odor source, surface roughness (e.g., grass, carpets) can either limit turbulent mixing very close to the substratum or enhance it a small distance above the substratum.

## 3 Manipulating training aid odor availability

Having discussed the foundational scientific principles that govern odor availability of a substance, variables impacting odor availability can now be discussed. The aim of this section is to discuss how temperature, surface area, containment, concealment, and set time can be manipulated to increase or decrease odor availability of a training aid. While not an exhaustive list, these factors may carry the most significance for canine trainers.

### 3.1 Temperature

The nonlinear relationship between vapor pressure and temperature is described by the Clausius-Clapeyron equation for pure substances and Raoult's Law for mixtures (66, 67). From this relationship, as the temperature increases the vapor pressure also increases. Increasing the vapor pressure also results in increasing the concentration of odor available in the headspace. While the canine community does not routinely use the formal terminology to describe the observation, the relationship between temperature and odor availability over time is well known within the community as most handlers and trainers observe the canine's finding the training aids more readily on a warm day vs. a cold one.

While vapor pressure is an inherent property that cannot be directly altered, the impacts of temperature can be capitalized on for training purposes. To increase the odor availability, one can work in “warmer” conditions. This may include running training exercises in the full sun, outdoors on warm days, or indoors on cold days. To decrease odor availability, one can work in “cooler” conditions. Cooler conditions may include running training exercises in shady areas, outdoors on cold days, or indoors on warm days. However, due to the hazardous nature of several training aids, they should not be artificially heated (e.g., placed on a hot plate) since hazardous gases or explosive reactions may be produced. Additionally, adding heat to human remains or biomedical detection dog training aids, such as patient samples or microbes, may permanently alter the training aid and its odor, thus compromising both canine detection of the training aid and the training aid itself.

### 3.2 Surface area

As previously discussed, evaporation and sublimation have been studied and modeled (28, 42, 68–70). Since mass flux is the rate at which the training aid sublimes/evaporates per unit of area, varying the surface area will directly impact the flux of material which in turn directly impacts the odor plume concentration. In this scenario, the surface area corresponds to the surface area of the training aid itself which may be the source material or the permeable area. For the purposes of surface area impacts alone, these two cases can be treated synonymously. Flux is material specific and constant, therefore increasing the surface area will result in increased odor availability and decreasing the surface area will result in decreasing the odor availability (16, 17).

**Figure 6:**
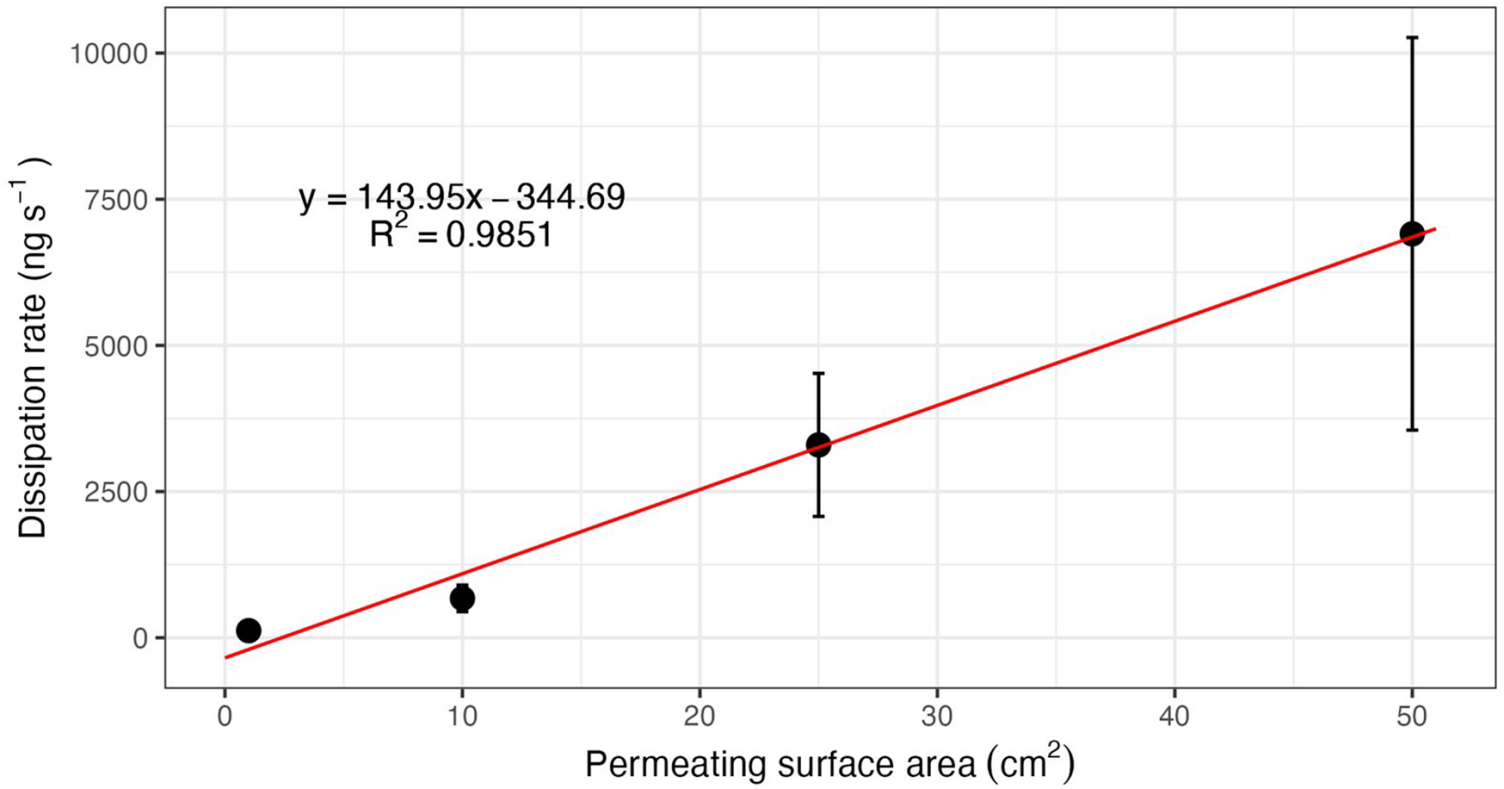
Linear relationship between permeable surface area and flux (dissipation rate). Reproduced with permission from Beltz, 2013 (16).

Often canine trainers have limited training aids and configurations, however, careful planning and packaging of the training aids in a variety of configurations can assist with training up and down a concentration ladder. For example, to increase the odor availability, one could use multiple training aids in the same location, spread the training aid out to cover more area, or use a training aid configured with a large permeable surface area (e.g., spread over a 10″ × 10″ polymer bag). To decrease odor availability, one could put out fewer training aids, contain the training aid into a smaller area, or use a training aid configured with a small permeable surface area (e.g., spread over a 1″ × 1″ polymer bag or odor restricting cap). Figure 6 illustrates the linear relationship between surface area (of either the training aid or the permeable surface) and dissipation rate of the training aid odor.

### 3.3 Containment

It is best practice to contain training aids in some form of primary containment (2). It is assumed that the flux from the source material greatly exceeds that of the permeation through the primary containment. This assumes a sufficient quantity of source material is placed in the primary containment for equilibrium to be reached. Thus, to increase the odor availability, one could remove the barriers to permeation (e.g., open a TADD® lid), use a thinner permeable material (e.g., selection of COMPS bag thickness), or use compatible containment materials that readily allow for diffusion. To decrease the odor availability, one could increase the barriers to permeation (e.g., multiple containment layers or bags), use a thicker permeable material, or use a less compatible containment material that minimally allows for diffusion. As discussed in the previous section, Fick's Law does incorporate a term for surface area, therefore increasing the surface area of permeable material will also increase the odor availability whereas decreasing the surface area will decrease the odor availability (71).

### 3.4 Concealment

For the purposes of this review, concealment describes how the training aid is hidden within the training environment. Concealment varies greatly and may include placement in a paint can for odor recognition test (ORT) scenarios, the gas cap of a vehicle for vehicle searches, or inside of a jacket pocket for person-searching scenarios. The purpose of the concealment is twofold: first to prevent visual detection of the training aid by the canine and handler and second, to replicate anticipated operational search needs. As previously described, as the distance from the source material increases, the odor concentration decreases due to dispersion, Figure 5b. This is nearly impossible to model due to the numerous variables, but some assumptions can be made based on the complexity of the concealment, the depth of the training aid within the concealment, and the airflow within the environment.

The complexity of a concealment will directly impact the odor availability because the more complex the concealment, the more likely there are multiple barriers to dispersion. For example, a training aid placed in a piece of luggage in a parcel search is significantly less complex than a training aid placed in a piece of luggage that is stacked on a pallet with several other pieces of luggage. As a general rule of thumb, odor availability can be increased through simple concealment and decreased with complex concealment.

Odor availability is also affected by the composition of the concealment material. The surface interactions between the odor profile of the training aid and the concealment material can lead to highly variable odor availability and persistence in the environment. Depending on the **adsorption** coefficient of the individual gas molecules of each odorant within the odor plume and the chemical composition of the concealment material(s) (whether that is a cardboard box, metal paint can, cloth backpack, or the carpeted trunk of a vehicle), training aid odors will interact with material surfaces via van der Waals forces (physisorption), covalent bonding (chemisorption), hydrogen bonding, and other intermolecular forces (72–75).

The depth of a training aid can be manipulated to increase or decrease the concentration of available odor. Training aids placed in concealment close to the surface have a shorter distance to disperse and experience more of the bulk flow that transports odors long distances before reaching a location that the detection canine can sample from, therefore there is an increased concentration at the sampling location. While training aids placed deep within a cabinet for example must disperse across a longer distance in which the concentration of odor is more diluted by the time it reaches the location in which the canine can sample (e.g., the cabinet door seam).

### 3.5 Air flow

Environmental air flows can drastically affect the concentration of odor experienced by canines during sampling events. Air speeds and directions, presence of convective flows (flows driven by temperature differences), presence of objects to disrupt flow, and position of hides with respect to the ground or floor can all influence how well mixed odors are in flow and the spatial positions, timing, and concentration of odors experienced by canines during a search in an odor plume.

Airflow within an environment can to a degree be manipulated. For example, fast airflows within an environment will produce a diluted concentration of odor within the odor plume whereas slower airflows have less dilution and therefore higher odor availability within the odor plume. Air speed is a delicate variable to balance as environments with minimal airflow can be exceptionally challenging for canine detection because dispersion is significantly reduced.

Unfortunately, air flow is more often difficult or impossible to control in a training space, especially outdoors. Furthermore, deliberately modifying air flow can have unexpected effects on the odor plume, since air flow is difficult to visualize and how it affects odor availability experienced by the canine is not well understood. For example, increasing air flow around a large object may concentrate odor in the wake of that object, rather than dilute it.

Generally, exposing canines to a variety of flow conditions during training will help to minimize the effects of air flow on detection success. These flow conditions can include: various wind speeds (still, breezy, very windy), wind directions (changing directions and straight-line conditions), varying humidity (which tends to influence where odor travels away from the substratum), areas that are flat or hilly, and areas that are clear or cluttered with objects or vegetation (which will affect how odor pools and collects in wakes). Additionally, the use of fans or HVAC system settings in a regular training space can create a novel challenge for canines used to certain training environments and can serve to alter the availability of odor. Importantly, these changes can also alter the spatial and temporal patterns of odor signals that dogs read to scent to source, making them more effective at this task in a wider variety of environments.

### 3.6 Set time

The time between the placement of the training aid in the concealment to the first detection canine performing a search is the set time. Anecdotally, a minimum of 30 min is used as a set time (2, 7). Since a training aid in concealment is unlikely to achieve equilibrium with its surroundings, in general terms, the longer the set time the higher the odor concentration because the flux is constant. Therefore, to increase odor availability longer set times should be used. Conversely, to decrease odor availability, reduced set times should be used.

## 4 Common training aid questions

*Can I increase the concentration of available odor by increasing the weight of the training aid?* The simplest answer is no. While a significant increase in weight typically corresponds to an increase in surface area due to sheer volume, manipulation of weight alone will not change the amount of available odor at equilibrium without the manipulation of other factors such as surface area, temperature, etc. Based on the findings of Giordano et al., and their study of the relationship between TATP vapor concentration and weight of TATP, it is recommended that, to avoid a significant decrease in available odor, the entire bottom of primary containment and a more than minimal depth of the bed of the training aid, be employed (29).

*What happens to odor availability if I increase the amount of training aid in primary containment?* Primary containment puts the training aid material into a small volume of space which allows the training aid to reach or nearly reach equilibrium. Since the equilibrium concentration for a training aid is constant, increasing the weight of material within the primary containment only results in equilibrium being achieved faster since there is a reduced air volume in the primary containment. You can, however, manipulate odor availability out of the primary containment by altering characteristics of the gas permeable material, i.e.*,* surface area, pore size, or nominal thickness.

*When it comes to detonation cord, can I increase the concentration of available target odor by increasing the length of the detonation cord used?* The simplest answer is no. It is generally accepted that much of the available odor is released via the cut ends of the detonation cord, therefore, while increasing the length of the detonation cord does increase the overall surface area of the training aid, the majority of the additional length is “sealed” by the cord plastic wrapping materials which are minimally compatible with permeation. However, this general acceptance requires further validation and study to be confirmed. Anecdotally, trainers have been known to suggest using multiple smaller lengths of cut detonation cord and or piercing the lengths of the cord randomly to increase the surface area of exposed training aid material.

*What is the set time for XX training aid?* Without knowing the temperature of the environment, volume of the concealment, surface area of training aid, etc. a specific time cannot be determined. This is also exponentially confounded by the addition of external turbulent air flows. In general, a minimum of 30 min is used as a set time for certifications (2). In training, if the training problem is particularly complicated (deep hide, complex concealments, etc.) or a cooler day than normal, the set time may increase. Alternatively, simple training problems (minimal concealment) and/or warm days may require less set time before a detection canine can successfully locate the training aid. Set times used in training should mimic how detection canines are used in operational settings.

## 5 Conclusions

**Figure 7:**
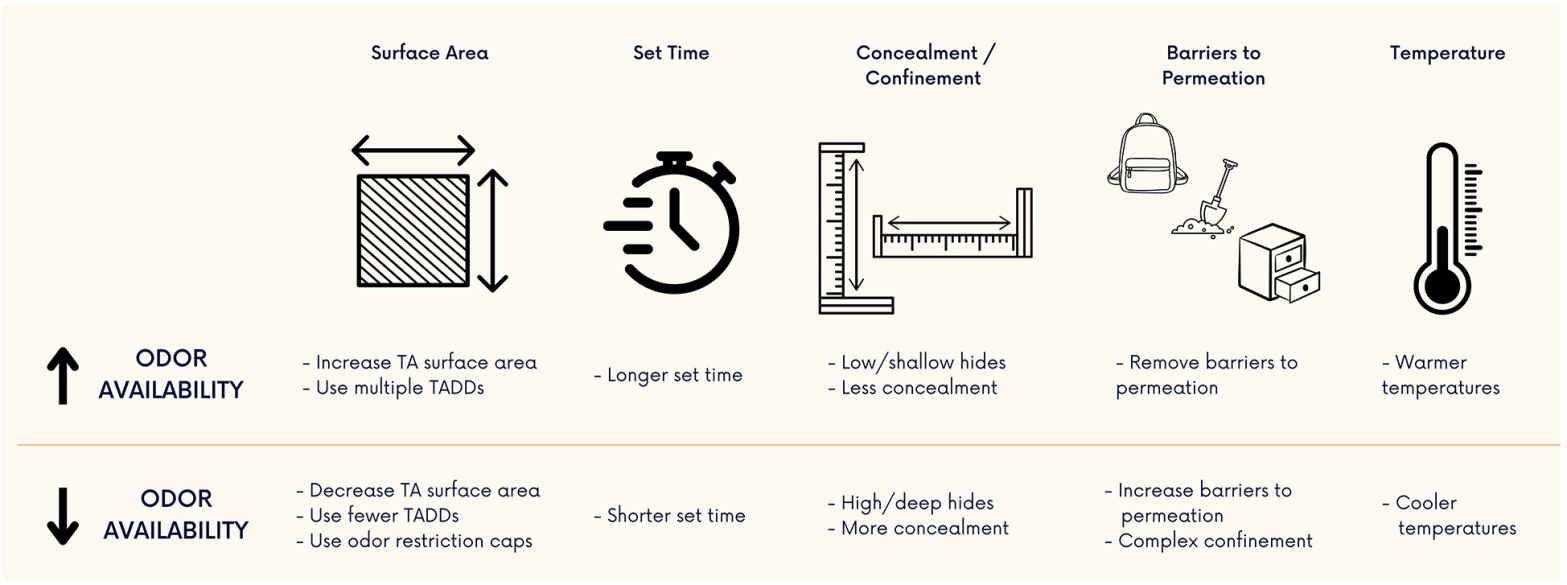
Methods of altering odor availability from source material. Odor availability can be manipulated by using some of these methods. Training aid (TA) odor can be increased or decreased by modifying the surface area, set time, degree of concealment/confinement, barriers to odor permeation, and temperature of the training environment.

Within this review we have shown that while the concentration of odor is typically unknown, it can still be manipulated. Several commonly encountered questions from the detection canine training community have been conglomerated and answered resulting in general guidance for training aid manipulation below (Figure 7):

1. To increase odor availability one can:
  a. increase the surface area of the training aid
  b. increase the set time
  c. decrease training aid hide complexity, concealment, confinement
  d. remove the barriers to permeation
  e. work in “warmer” temperature conditions
2. To decrease the odor availability one can:
  a. decrease the surface area of the training aid
  b. decrease the set time
  c. increase training aid hide complexity, concealment, confinement
  d. increase the barriers to permeation
  e. work in “cooler” temperature conditions

One way for canine trainers to ensure that they are presenting as many different odor availability scenarios to their dogs as possible is to incorporate variability into their training regimens. By training in different weather conditions, using a variety of containment and concealment options, and changing set times, dogs will become more dynamic in their detection abilities and ultimately more reliable in operations.

Future work on integrating and harmonizing operational canine detection scenarios, terms and definitions, training methodologies, and record-keeping with canine detection science to include applied research, development, test, and evaluation will help both end-users and scientists develop a shared language and ultimately advance and enhance canine detection performance.
